# Barriers to Behavior Change in Parents With Overweight or Obese Children: A Qualitative Interview Study

**DOI:** 10.3389/fpsyg.2021.631678

**Published:** 2021-03-26

**Authors:** Katrin Ziser, Stefanie Decker, Felicitas Stuber, Anne Herschbach, Katrin Elisabeth Giel, Stephan Zipfel, Stefan Ehehalt, Florian Junne

**Affiliations:** ^1^Department of Psychosomatic Medicine and Psychotherapy, Medical University Hospital Tuebingen, Tuebingen, Germany; ^2^Public Health Department of Stuttgart, Stuttgart, Germany; ^3^Department of Psychosomatic Medicine and Psychotherapy, Otto von Guericke University Magdeburg, Magdeburg, Germany

**Keywords:** readiness to change, parental role, obesity, overweight, children

## Abstract

Overweight and obesity among children and adolescents are global problems of our time. Due to their authority and role modeling, parents play an essential part in the efficacy of prevention and intervention programs. This study assessed the barriers that parents of overweight/obese children face in preventive and interventional health care utilization. Sixteen parents were qualitatively interviewed. A content analysis was performed, and barriers to change were allocated to their stage of change according to the transtheoretical model. Among the main barriers is the underestimation of health risks caused by overweight/obesity in association with diminished problem awareness. Parents seem not necessarily in need of theoretical knowledge for prevention and interventions. They do however need support in evaluating the weight status of their child and the knowledge of whom to turn to for help as well as specific and hands-on possibilities for change. The results extend past studies by adding specific barriers to change that parents commonly experience. Possibilities to address these barriers, e.g., through trainings at the pediatric practice or adoption of conversation techniques, are discussed. Future studies might identify subgroups experiencing specific barriers and thus be able to address these in an individualized way.

## Introduction

Overweight and obesity among children and adolescents are global problems of our time (World Health Organization (WHO), 2014). In Germany, about 15% of children and adolescents are affected by overweight and about 6% by obesity (Kurth and Schaffrath Rosario, 2007; Schienkiewitz et al., 2018). Many children and adolescents stay overweight/obese during their life span, leading to an increased risk for somatic and mental health comorbidities, high health expenditure, and even a shorter life expectancy (Wardle and Cooke, 2005; Giel et al., 2012; Linder et al., 2014; Effertz et al., 2016). Advancing intervention as well as prevention programs seems therefore inevitable.

Evidence suggests the importance of starting intervention and prevention programs at an early age. Childhood overweight and obesity in preschool children have dramatically increased in the last decades (De Onis et al., 2010). A longitudinal study by Shankaran et al. (2011) showed a two-fold risk for being overweight/obese at the age of 11 when the body mass index (BMI) values of preschool children were above the 85th percentile. In light of the strong association between childhood obesity and adulthood obesity (Simmonds et al., 2016), interventions should already start at the preschool level. Expanding intervention and prevention programs to other contexts such as childcare settings or the family/parents is also important (Birch and Ventura, 2009).

Due to their authority and role modeling, parents play an essential part in the efficacy of prevention and intervention programs targeting their children. For example, Sigmund et al. (2018) reported on parents' key role in forming children's health behaviors. The American Heart Association also refers to parents as “agents of change” for their children (Faith et al., 2012). Both emphasize the defining role of parents for the success or failure of or even participation in weight management and/or lifestyle interventions for overweight and obesity. Perceived barriers of parents to prevention and intervention seem therefore of great importance.

Studies to date have shown only parts of the complex barriers parents can experience. Schmied et al. (2018) for example identified lack of family support and scheduling difficulties as barriers for parents to participate in the specific intervention program that was offered. An interview study by Nepper and Chai (2016) identified parental barriers to healthy eating, but their sample was not overweight/obesity specific and focused solely on nutrition. Concerning portion sizes, parents seem to be mostly concerned about their children not getting enough food at mealtimes and not sure about the appropriate amount of food, thus being hesitant to restrict portion sizes (Eck et al., 2018). A systematic review of parental perceptions regarding healthy behaviors for preventing overweight/obesity in children did extract some barriers of parents (Pocock et al., 2010). It remains unclear, however, if these barriers also apply to parents of children who are already overweight/obese. In summary, most of the studies until now have not looked at overweight-/obesity-specific groups and/or only looked at specific aspects of potential barriers (e.g., nutrition or physical activity), thus omitting the bigger picture.

The present study therefore aims to qualitatively assess the barriers of parents with overweight and obese children to preventive and interventional health care utilization in a phenomenological qualitative research approach (Groenewald, 2004). Due to the early occurrence of overweight and obesity, the strong association with high weight later in life, and the defining role of parents at this age, parents of preschool children were selected to participate in the current study. An emphasis was put on including parents who belonged to at least one of three risk groups for childhood overweight/obesity as determined in a representative study in Germany: overweight/obesity of at least one parent, a migration background, and low family income (Kurth and Schaffrath Rosario, 2007). Evidence from international studies also highlights the importance of these risk factors (Eagle et al., 2012; Furthner et al., 2017; Heslehurst et al., 2019).

For a guiding theory, the results are allocated according to the stages of change of the transtheoretical model (TTM; Prochaska and Diclemente, 1982; Norcross et al., 2011) of behavior change. The TTM was chosen in its common representation with four stages of change on the way to changing health behaviors (e.g., in the case of overweight/obesity): (1) precontemplation, where problem awareness is absent and there is no intention for change; (2) contemplation, where problem awareness is present, intention to change is being evaluated or present, and no action has been taken yet; (3) action, where active effort is made to change the problematic behavior; and (4) maintenance, where problematic behaviors have been changed and there is an active effort to maintain change.

Evidence supports the efficacy of interventions tailored to the stage of change of individuals for a variety of behavioral problems including eating behavior and physical activity (Marcus et al., 1998; Klöckner and Ofstad, 2017; Krebs et al., 2018; Teng et al., 2020). In the childhood overweight/obesity literature, the TTM has been applied as a helpful conceptual framework in a variety of quantitative studies (Rhee et al., 2005, 2014; Sealy and Farmer, 2011; Giannisi et al., 2014). It therefore provides an important framework for the identification of specific barriers within the different stages of change. Assembling the barriers according to these stages enables the derivation of a model of barriers to change of parents with overweight and obese children and promotes the development and advancement of individualized prevention and intervention programs.

## Materials and Methods

### Sample and Sampling Strategy

Parents presenting their children at the school enrollment medical examination within a 12 months' time span in a large German city were asked to participate in a large quantitative study. Out of 5,017 parents, 1,320 participated in the quantitative study, of which 113 parents had overweight or obese children (Junne et al., 2016). Ninety-four parents consented to be approached for this qualitative study. Inclusion criteria were that parents had at least one child with overweight (BMI > 90th percentile) or obesity (BMI > 97th percentile) (Kromeyer-Hauschild et al., 2001) and had sufficient German language skills to be interviewed.

The school enrollment medical examination was chosen for recruiting parents since it is mandatory in Germany for their children and therefore provides the opportunity to recruit in an unbiased setting in comparison to, e.g., a pediatrician practice in which parents might only present themselves if overweight/obesity of their children is already a concerning topic for them. No criterion for sampling saturation was set, and all parents of overweight/obese children could participate. To ensure heterogeneity of the final sample, when contacting potential participants for the second time, emphasis was put on trying to contact and invite families in which parents belonged to at least one of three risk groups for childhood overweight/obesity: (1) at least one parent is overweight/obese themselves, (2) at least one parent has a migration background, and (3) the net household income per month is below the 25th percentile (<2,000€) (Kurth and Schaffrath Rosario, 2007).

### Measures

Parents were interviewed according to a semi-structured interview guideline addressing six core topics concerning the overweight/obesity of their children: (1) problem awareness, (2) possibilities of the parental role, (3) utilization of preventive and interventional actions, (4) barriers to change, (5) stigma and social network, and (6) prevention. The interview guideline consisted of 25 open main questions with up to seven side questions each for deepening the respective topic (see Supplementary Table 1). It was developed by the study team, pilot-tested, and adapted with regard to practicality, comprehensibility, and specification of questions. Since the interview was semi-structured and conducted on the phone, researchers' characteristics potentially influencing the study were kept to a minimum.

### Procedure

Ethical approval was obtained from the ethics committee of the medical faculty of the University of Tübingen (no. 509/2013BO1). Parents were informed about the study and invited to participate. Participating parents were given a short demographic questionnaire and consented to participate in the study and to be contacted by phone for an interview in the near future. Only one parent per family could participate. The interview length ranged between 13 and 69 min, with a mean duration of 40 min, and was predominately dependent on the elaborateness of parents' answers. All interviews were recorded and subsequently sent to a transcription company. The anonymized transcripts were analyzed by the study team.

### Qualitative Analysis

A primarily inductive qualitative content analysis according to Mayring (2014) was performed. This method was chosen due to the perceived shortcomings of previous studies that applied predefined categories/criteria that parents could rate as important/unimportant. This inductive approach accounts for the diversity and individuality of barriers parents were expected to experience.

To ensure trustworthiness and credibility of the data analysis, it was triangulated between different members of the study team: All interviews were independently analyzed by two members of the study team (team 1). In a first step, categories were deduced from three sufficiently long (>30 min) randomly chosen interviews by paraphrasing, with generalization to the required level of abstraction and reduction. The categories identified by team 1 were discussed in the study team and combined to a category system which was applied to two other interviews of the sample by two other instructed members of the study team (team 2) as well as team 1. The resulting category system was discussed in the study team, adapted, and applied to three additional interviews for refining. Disagreements were discursively solved. At this point, reproducibility was achieved, and no new categories could be deduced. The seven interviews that were used to develop the final category system were about 44% of the total material for analysis, therefore ranging within the 10–50% Mayring (2014) plans for revision of categories and rules. The final category system was subsequently applied to the remaining transcripts. All of the codification was performed using the qualitative data analysis software “MAXQDA 11” (Verbi Software, 2019).

After the category system was applied to all interviews, the content of each main category was analyzed by extracting the quintessence, discussing and interpreting it in the study team, and evaluating it as either a resource or a barrier to change. The derived barriers were grouped into the stages of change according to the TTM; hence, the final model of perceived barriers to behavior change was derived. After the model was established, a cross analysis of the material from all categories was performed to ensure fit.

## Results

### Participants

**Figure 1:**
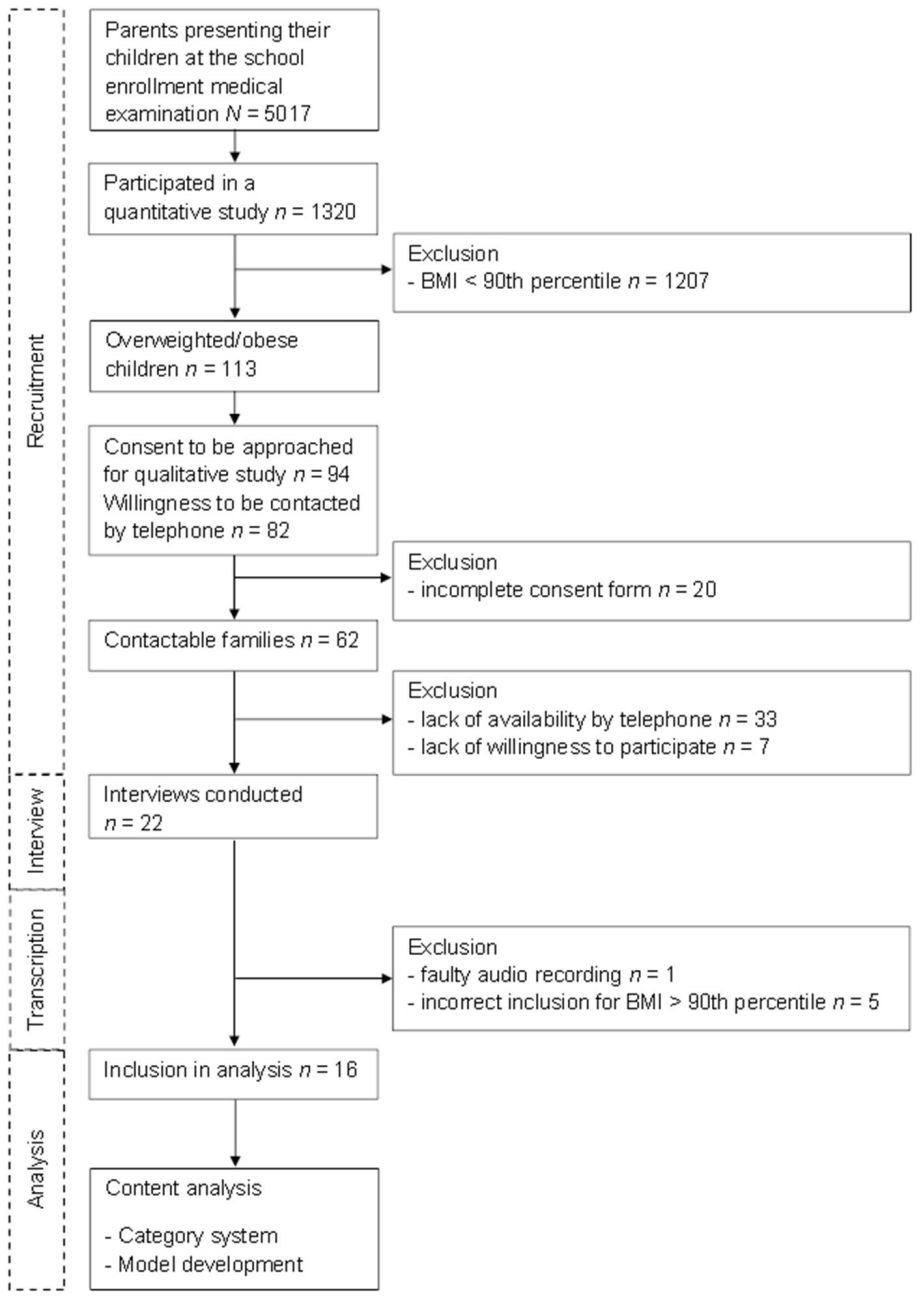
Flow diagram of the study enrollment and analysis process.

Out of the approached families, 62 fulfilled the criteria to be contacted by phone and asked to participate, and 22 parents were interviewed, of which five had to be excluded because their child's BMI was less than the 90th percentile, and one interview was excluded because of technical problems. Finally, 16 interviews could be included in the final analyses. The flow diagram of the study enrollment and analysis process can be found in Figure 1.

The demographics of participating parents and their overweight/obese children are presented in Table 1. Notably, all parents belonged to at least one of the risk groups for childhood overweight/obesity: overweight/obesity of at least one parent, migration background of at least one parent, or total household income at less than the 25th percentile (<2,000€/month after deductions).

**Table 1:** Demographics of participating families.

	***N***	***M (SD)***
**Overweight/obese children**		
Gender		
Female	12	
Male	4	
Age		5.1 (0.3)
BMI percentile		95 (3)
Overweight	12	
Obesity	4	
**Participating parents**		
Gender		
Female	14	
Male	2	
Age		
Mother		38.7 (4.0)
Father		41.5 (0.5)
Migration background		
None	5	
One-sided	4	
Both	5	
Not specified	2	
Net household income per month		
<2,000€	3	
>2,000€	12	
Not specified	1	
Weight category		
No overweight/obesity	6	
Overweight	8	
Obesity	2	
Family weight		
One-sided overweight/obese	9	
Both overweight/obese	6	
Not specified	1	

*BMI, body mass index; M (SD), mean (standard deviation)*.

### Category System

The final category system of the qualitative analysis consisted of 25 main categories that are listed in Table 2.

**Table 2:** Main categories of the final category system.

- Handling strategies/perceptions of weight - Perception of children's weight - Level of information - Migration/culture - Environmental conditions: society/politics/infrastructure
- Upbringing - Role modeling of parents - Habits/comfort zones - Causes/explanations of overweight/obesity by parents - Shame and fault - Time - Financial resources - Mental health
- Physical health - Other barriers - Other resources/prospects - Measures planned so far - Third-party recommendations ignored so far - Measures conducted so far
- Experiences with pediatricians and general practitioners - Experiences with daycare facilities/nursery schools/schools - Experiences with other places - Experiences in conducting previous measures - Potential contact persons/care providers

### **Model of Barriers to Behavior Change of Parents With Overweight and Obese** Children

**Figure 2:**
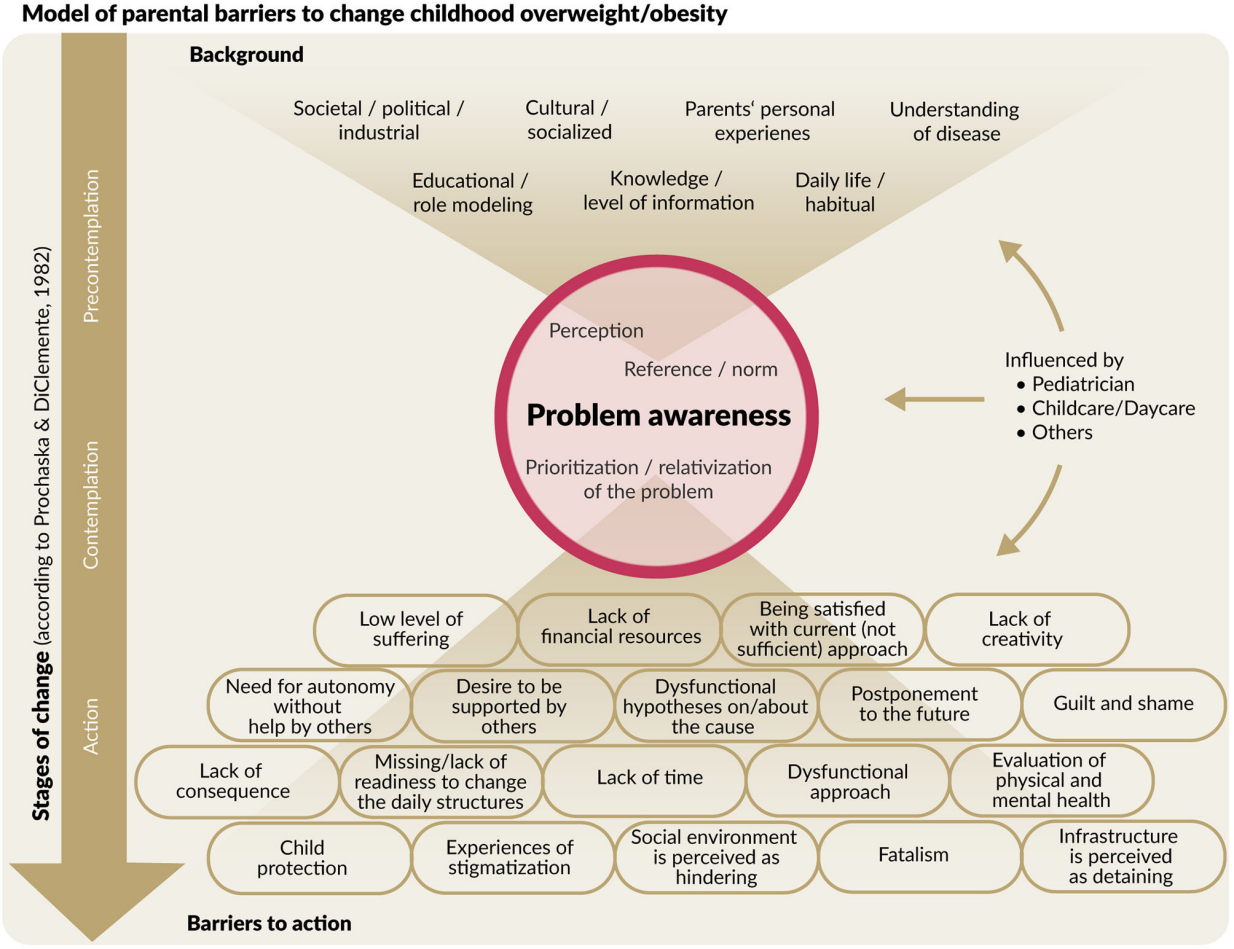
Final model of parental barriers to change in their children's overweight/obesity. Stages of change refer to the transtheoretical model (TTM) by Prochaska and Diclemente (1982).

The final model is presented in Figure 2. The background section contains variables representing background information or factors influencing parents' views on the subject of overweight/obesity in the stage of precontemplation (and beyond that). Each of these factors can enhance or hinder problem awareness, although, in the presented model, the focus is on barriers. Problem awareness is the key stage leading to action and can thereby in itself be a barrier to changing children's overweight/obesity. Barriers to action are the factors mentioned by parents who are aware of the problem but are not (adequately) taking action against it. These also represent the action stage of the TTM.

Unsurprisingly, since all of the interviewed parents had children who were overweight/obese, no barriers to maintaining achieved change (i.e., weight normalization or weight loss) were explicitly mentioned; thus, the maintenance stage of the TTM was not represented in our final model. However, a lot of the barriers experienced in the action stage, such as lack of time, desire to be supported by others, or a hindering infrastructure, might apply to the maintenance stage as well. In the following, the different sections of the final model as well as some exemplary barriers are presented. A list of exemplary quotes of all identified barriers can be found in Supplementary Table 2.

#### Background

This section contains areas affecting the knowledge and/or living environment of the interviewed parents. The “knowledge/level of information” category, for example, refers to heterogeneous statements of parents either negating the need of information on childhood overweight/obesity or showing satisfaction with their knowledge even if it was false. Example statements of parents were as follows:

“[…] no, I am informed. Well informed.” (Interview Mother no. 12, IM12)

“Having 2, 3 or even 5 kg more at the moment is okay, I think.” (Interview Father no. 3, IF3)

Although gaining 5 kg of weight might be okay for an adult, it can mean going from the 50th to >90th percentiles for a 5-year-old. Some parents also stated their need of information on the topic to determine if weight could be a problem.

#### Problem Awareness

A key factor in the process of change is being aware that something is a problem. It takes us from the stage of precontemplation (not being aware of the problem and not taking any action concerning the problem) to the contemplation stage (being aware of the problem and thinking about addressing it). Even though there are also a lot of barriers to taking action, without problem awareness, there will be no change.

In the present model, three categories were derived that served as barriers to the development of problem awareness: “perception,” “prioritization/relativization of the problem,” and “references/norms.” In the perception category, parents described not perceiving their child's weight as a problem or being “blind” of the problem concerning their own child. The following statements were examples:

“… no one of us is overweight” (IM11) or “I would pay attention to it, if he was overweight” (IM15).

In the “prioritization/relativization of the problem” category, parents were minimizing the overweight/obesity of their children as well as underestimating the health risks that can follow. A very common relativization of parents was the thought that even if their children were overweight right now, they would “grow out” of it, which seemed to be confirmed by some doctors and other third parties (e.g., other parents). This reasoning continued in the “references/norms” category, where parents mainly used comparisons with other children and/or clothing sizes to relativize the weight of their child (“broader shoulders,” “not as heavy as the heaviest child of his/her age,” “not as long as wide,” “can still wear normal pants,” and “does not need special clothing sizes”) or found it difficult to determine if the weight of their child was too much.

“Most of her friends are the total opposite. These small, really, really, thin girls. It is therefore difficult to compare her to most of the others. She is relatively tall for her age. You notice she is sturdy in comparison to a lot of the others. But that's what I say, direct comparisons are often to the really, really thin girls and that is really difficult to compare.” (IM12)

“You see other children at the playground or at the swimming pool and think: My child is in fact way too thin.” (IM11)

Overall, the described strategies to determine overweight/obesity of their children reported by parents often included references and comparisons to others. Weighing their child or other objective measures were rarely reported. Word choice of parents for describing the weight of their children was often relativizing (“not really overweight,” “not dramatic,” “of heavy build,” and “minimally overweight”). The description of seemingly thin children, however, was negatively biased and perceived as a bigger problem than overweight.

#### Barriers to Action

In this category, parents' barriers to actively doing something to change the overweight/obesity of their children are represented. These barriers imply problem awareness of overweight/obesity in childhood and refer to aspects why action was not possible or is going wrong. The complete list of identified barriers can be found in Figure 2. Some are exemplarily illustrated in the following.

#### Dysfunctional Hypotheses About the Cause

Although interviewed parents named poor nutrition and insufficient exercise as the main causes of overweight/obesity in general, a great deal reported physique and height as the cause of overweight/obesity of their respective children. In succession, they did not deem it necessary to change their families' nutrition and/or exercise level.

#### Dysfunctional Approach

In this category, parents or other caregivers take inadequate action that hinders change. Most described barriers thereby refer to actions concerning the nutrition of the child. Monitoring the intake of sweets was a frequent measure and frequently the only one taken for better nutrition. Other aspects included having no control about other caregivers, giving inadequate snacks as a standard, using food as a reward, or simply monitoring the weight as a sole measure.

“They always get candy at their grandparents' house. [Name of child] has a sensitive stomach, if she really eats too much candy, it happens that she throws up. […] I asked my father to stop doing this. Or giving her just one piece.” (IM20)

“We waited for this [weight normalization].” (IF3)

#### Social Environment and Infrastructure

These categories refer to the continuous availability of food around environments that families frequently visit (e.g., an ice cream store is right next to their favorite playground) or the living/care situation (e.g., the family lives on the fourth floor and the child is not (yet) able to play in the yard by itself).

#### Protection of the Child and Evaluation of Physical and Mental Health

Although action was taken, parents reported on limiting changes and not talking to their children about the measures taken in order to protect their mental health. Avoidance of shame, guilt, self-stigmatization, and the prevention of eating disorders were the reasons for parents to do so. Parents also reported on prohibiting the pediatrician or other third parties from speaking about weight, nutrition, and exercise in front of their children.

Interviewer: “Has he [the pediatrician] tried to explain [daughter] the association between weight and nutrition before?” Mother: “Not directly, but I would have stopped that anyway.” (IM21)

“When I tell her, you can only eat this or that or you can only eat that much … I think that will make it even harder for the child and at her age, it really puts a strain on her.” (IM11)

#### Desire to Be Supported by Others

Prominent was also the desire to receive more support by others, especially professional support by, e.g., the pediatrician, the general practitioner (GP), and the daycare or childcare facility. The pediatrician was of particular interest, with parents hoping that he/she would take responsibility, talk to them about the weight of their children, and tell them what to do or, in case he/she does not know, refer them to the correct information center or the like. When conversations about weight management did take place, parents perceived them to be short and be held in passing. They instead would like to have detailed guidance on childhood weight development.

“I would expect our pediatrician to help us and if he doesn't know how, that he refers us to the right place.” (IM17)

#### External Influences

Answers by parents throughout all stages of the model frequently reported on influences of third parties. These were predominately other parents or other family members (i.e., grandparents), the pediatrician, and the daycare or childcare facilities. These third parties had manifold influences on interviewed parents by either enforcing that parents are doing everything just fine and there are no problems, lack of taking responsibility and the expectancy of parents that third parties would say something if indeed the weight of their children was a problem. There were also great influences during the stage of problem awareness and action. Third parties are therefore represented in the model as contributing influences on all three stages.

## Discussion

The present study is, to the best of our knowledge, the first qualitative study identifying barriers to behavior change in parents of preschool children with overweight/obesity that comprised a sample of parents with at least one of three identified risk characteristics for developing overweight/obesity. Among the main barriers this study identified is the underestimation of health risks caused by overweight/obesity in association with deficient problem awareness. The results are in line with past studies showing that parents frequently do not detect overweight/obesity of their children (Manios et al., 2009; Warschburger and Kröller, 2012; Rietmeijer-Mentink et al., 2013; Lundahl et al., 2014; Hochdorn et al., 2018), which hinders problem awareness.

The present study extends these findings by suggesting that despite being informed by the pediatrician about the overweight/obesity of their child, some parents do not develop problem awareness. This is supported by a study by Dawson et al. (2014) showing that parents were able to give an account of their child's elevated weight when being informed by the pediatrician about it. However, they could not recall much about further information on overweight/obesity or any advice provided by the pediatrician. Information about the problem by an expert seems therefore not sufficient to elicit problem awareness. Even after parents are aware of the child's overweight/obesity being a problem, there are many potential barriers left.

One of these barriers refers to the discrepancy between perceived causes of overweight/obesity in general and causes of their respective child's overweight/obesity in parents' interviews. Causes of overweight and obesity in general were primarily seen in nutrition and exercise/activity. Causes of the own child's overweight/obesity, however, were primarily seen in causes that parents have no influence on such as physique, height, or a “slow gastrointestinal tract.” This implies that overweight/obesity in general might be changed since it originates from causes parents have an influence on. Overweight/obesity however might not seem changeable in the eyes of parents in case of their own children.

An important reason for trying to aim for change in addressing overweight and obesity in children is, however, that they are likely to persist into adulthood, especially for high-risk groups (Singh et al., 2008). The importance of prevention and early interventions is therefore commonly advised, e.g., in the German guidelines for diagnostics, treatment, and prevention of childhood overweight and obesity (Reinehr et al., 2008; Arbeitsgemeinschaft Adipositas Im Kindes-Und Jugendalter (Aga) et al., 2019). Regardless, parents in the present study frequently postponed this problem to the future or showed readiness to postpone it. A study by Eli et al. (2014) also showed parents being aware of their children's overweight/obesity but perceiving it as a problem of the future (namely, at school age) when factors such as bullying, social exclusion, and changes of mood and behavior could occur.

The findings of Eli et al. (2014) are therefore in line with our finding about parents being oblivious to the health risks of overweight/obesity. Parents seem more likely to take action only when other areas of life such as social functioning or mood were affected by overweight/obesity. In sum, according to our findings, parents seem not necessarily in need of theoretical knowledge of factors for prevention and intervention such as nutrition and activity in general. They do however need support in evaluating the concrete weight status of their respective child, knowledge of whom to turn to for help, and specific and hands-on possibilities and guidance for change.

Concerning barriers to action, parents in the present study were missing concrete ideas as well as contact persons with which practical problems and barriers could be discussed. Physicians (GP or pediatrician) were the preferred professionals of parents for broaching the issue of overweight/obesity. Among the main wishes parents had for their pediatrician were for him/her to be their main contact person concerning their children's weight, him/her to address the weight issue, and him/her to give detailed guidance about options for action. Examinations and follow-ups should also be done by the pediatrician in the parents' eyes, i.e., accompanying the process of weight normalization. These wishes are in line with existing literature showing that the stage of change of parents depends on whether the physician describes the child's weight as a problem or not and if a concrete course of action was recommended (Rhee et al., 2005, 2014). In the present study, parents only differed in whether or not they favored the inclusion of the children themselves into the guidance by the pediatrician.

In order to fulfill these wishes of parents by the physician, it seems essential to provide specialized training courses concerning childhood overweight/obesity. A study about primary care clinicians' views of treating childhood obesity also points to a lack of resources and ill-equipped GPs and practice nurses concerning this topic (Walker et al., 2007). This training would not need to be exclusive to the pediatrician or GP but might include practice nurses or other health care professionals and could therefore enable individualized counseling (Sastre et al., 2019).

One possibility to strengthen the parents' readiness to change during individual counseling sessions, especially after identifying so many barriers to change, could be the use of conversation techniques that enhance motivation and willingness to change such as motivational interviewing (Miller and Rollnick, 2012; Junne et al., 2019). This technique originates in the area of substance abuse disorders and was developed to address highly ambivalent patients. It has since been widely applied for different mental disorders such as depression and eating disorders as well as for enhancing treatment commitment (e.g., medication adherence) in GP practices. A variety of studies showed the effect of motivational interviewing to enhance commitment and further readiness to change (Hettema et al., 2005; Rubak et al., 2005; Azami et al., 2020; Li et al., 2020).

The approach of such counseling sessions should be positive and resource based to avoid the mentioned barriers of shame, guilt, and self-stigmatization. Counseling topics could include nutrition and exercise, which have been frequently mentioned as causes of overweight/obesity. Other topics mentioned by parents of the current study when asked about important topics that should be addressed by counseling are body image, mood, impulsive eating, and stress eating. Other studies also identified family challenges and conflicts as well as poor sleep and advertising as relevant topics to address (Brogan et al., 2019).

Interestingly, advertising or media usage was not identified as barriers in the present study. In the literature, however, reduced activity was associated with more media consumption such as watching TV (Kleiser et al., 2009; Thundiyil et al., 2010). Media has also been shown to have negative influences through advertisements, especially in the food sector where there is an association between increasing advertisements for predominately energy-dense foods and food consumption of children (Boyland and Whalen, 2015). This might indicate an additional barrier to change that the interviewed parents of this study were not aware of and that might be addressed in individualized counseling sessions.

The present study has some limitations that should be mentioned. First of all, the cross-sectional design does not allow for sequential or even causal arrangement of barriers to change in childhood overweight/obesity. The established model however provides an overview of potential barriers to change at different stages of change. Furthermore, it remains to be determined if all of the identified barriers are of equal importance or frequency of occurrence. Future studies might be able to identify subgroups more likely to experience certain barriers and thus be able to address these barriers in an individualized way. The results of this study also inform a large cluster randomized controlled trial targeting families with overweight/obese children/adolescents [STARKIDS, Universal Trial Number (UTN): U1111-1254-9536].

## Data Availability Statement

The raw data supporting the conclusions of this article will be made available by the authors, without undue reservation.

## Ethics Statement

The studies involving human participants were reviewed and approved by Ethics committee of the medical faculty of the Eberhard Karls University of Tuebingen. The patients/participants provided their written informed consent to participate in this study.

## Author Contributions

KG, SZ, SE, and FJ contributed to the conception and design of the study. SD, FS, AH, and KZ substantially contributed to the acquisition of data for the study. SD and FS performed the analysis. KZ and AH wrote the first draft of the manuscript. All authors contributed to manuscript revision and read and approved the submitted version.

## Conflict of Interest

The authors declare that the research was conducted in the absence of any commercial or financial relationships that could be construed as a potential conflict of interest.

## Abbreviations

BMI: Body mass index
GP: General practitioner
IM[no.]/IF[no.]: Interview mother [no.]/interview father [no.]
TTM: Transtheoretical model.
