# Supplementary material for: Barriers to Behavior Change in Parents With Overweight or Obese Children: A Qualitative Interview Study
Source: Front Psychol. 2021 Mar 26;12:631678. doi: 10.3389/fpsyg.2021.631678 (PMC8034266; doi:10.3389/fpsyg.2021.631678)
Supplement: Supplementary file 1 [file Table_1.DOCX]

**Interview guideline for parents of children with overweight or obesity**

**Introduction**

- Thank you for participating in this preschool child health study!
- You have already completed a questionnaire on this subject.
- Now we would like to determine your views, attitudes, opinions, and thoughts about weight development in preschool children in the form of telephone interviews.
- You have been randomly selected for this interview. The interview will last about 30-45 minutes.
- The compensation for this is 30 euros.
- In principle, would you be interested in participating in this interview?
- Is it convenient for you now / Can you talk in peace now or would you like to make another appointment?

**When participating**

- This interview will be recorded on tape and transcribed.
- All personal information that could be used to draw conclusions about you will be deleted, which means your data will be treated completely anonymously.
- As I said, it is about your opinions, views, and experiences. There are no right or wrong answers.
- If you have any questions or have not fully understood something, please ask at any time.

**1. Problem awareness**

**How do you currently evaluate your child's weight situation?**

- How do you evaluate it compared to peers?
- How do you measure this?

**What is your opinion of childhood obesity?**

- Do you think being overweight changes a child's self-esteem positively or negatively?

**How would you describe your family's or culture's view of childhood overweight?**

- Do you think your family/culture has a different view of childhood overweight than the general population? Which one?
- How would you describe your own level of information about nutrition and weight?
- Do you have any need for information?

**2. Role potential**

**What do you think you can do to help your child achieve weight normalization?**

**Do you think of yourself as a role model for your child?**

- Do you think you are a role model for your child in terms of exercise and eating style?
- Would you be willing to change your behavior and habits regarding nutrition (cooking) and exercise to help your child achieve weight normalization?

**3. Utilization behavior of prevention and intervention measures**

**Have you ever searched for information on childhood overweight?**

- If yes: Where did you find it?
- If no: If you need information on childhood overweight - where do you look for information?
- Who can you contact personally for practical support/help?
- Do you think it would be helpful for you to have practical help?
- Who would you talk to about this issue?

**If you have not looked for information before, what has held you back?**

**If you have looked for information before, what were the hurdles/obstacles?**

**Who do you think is the best person to contact about problems with childhood overweight in the community/health care system?**

**Where do you think you can get help dealing with your child's overweight?**

- What kind of help do you expect to get when you look for it?

**Have you ever sought help in dealing with your child's overweight?**

- If no, why not?
- If yes, where did you try to get help?
- If yes, what were you advised/recommended to do?
- What specific areas of life were considered for change?

**Did you try to implement the actions recommended, if any?**

- If yes, which ones?
- If yes, with what success?
- What changed afterwards?
- Were there any specific changes in your child's behavior? If yes, which ones?
- Did you make any specific changes in your behavior? If yes, how?
- Was there visible success regarding weight or medical values?
- If you were not successful with the measure - what do you think was the reason?

**If you were to take measures, which ones would that be?**

- What would make it difficult for you?

**4. Barriers**

**What has prevented you from seeking information or help regarding your child's overweight?**

**If you have sought help, have there been any barriers to doing so?**

- How stressful do you and your child find being overweight?

**From your perspective, how difficult is it to contact help centers?**

**What might be reasons for not contacting a help center?**

- What would prevent you from contacting a help center?

**What other protagonists can do something to prevent obesity?**

**Do you have feelings of shame about your child being overweight?**

- If yes, in which situations?

**What offers would be important?**

**5. Stigma and social contacts**

**Do you have the impression that your child is sad/stressed more often because of being overweight?**

- Does your child pull back (socially) because of being overweight? For example, does he/she not like to play with other children?

**Do you think that your child - if he/she remains overweight - will have worse chances than others at school and at work?**

**Optional appendix**

**6. Prevention**

**In your opinion, what would be the best way to prevent a child from becoming overweight?**

- What can parents or the kindergarten do to prevent overweight?

**What would you generally advise parents whose child suffers from overweight?**

**If you could wish for some offer that does not exist, but that should exist, what can you think of?**

**What should change?**
