# Supplementary material for: Barriers to Behavior Change in Parents With Overweight or Obese Children: A Qualitative Interview Study
Source: Front Psychol. 2021 Mar 26;12:631678. doi: 10.3389/fpsyg.2021.631678 (PMC8034266; doi:10.3389/fpsyg.2021.631678)
Supplement: Supplementary file 2 [file Table_2.DOCX]

*Supplementary Table 2 Exemplary quotes of the identified barriers*

| **Barrier** | **Quote** |
| --- | --- |
| *Background* |  |
| Societal / political / industrial | ‘...all of this ‘children food’... If I go shopping with my daughter and she sees that cute rabbit on the packaging, she will like it no matter what is in it.’ |
| Cultural / socialized | ‘Well, if you look at the Germans, every other person is overweight. I think it has become...I don’t know, I personally think it is horrible but I think the culture...it’s a norm. Everyone knows it but nobody takes action against it.’ |
| Parents’ personal experiences | ‘I have to look after it myself and exercise and I also like eating...’ |
| Understanding of disease | ‘Where does overweight start? What marks overweight at that age? I think that is very difficult.’ |
| Education / role modeling | ‘I have a lot of children in my kindergarten that don’t pay attention, that, in my opinion, have more massive problems with their weight, the children. But I cannot tell the parents: Pay more attention to the nutrition of your child. That is not my style.’ |
| Knowledge / level of information | ‘[…] no, I am informed. Well informed.’ |
| Daily life / habitual | ‘Just regular habits...for him to learn this...to learn this lifestyle from an early age and practice it.’ |
| *Problem awareness* |  |
| Perception | ‘… no one of us is overweight’ |
| Prioritization / relativization of the problem | ‘I think her figure is normal. It’s only her belly. For my child. Just the belly.’ |
| Reference / norm | ‘You see other children at the playground or at the swimming pool and think: My child is in fact way too thin.’ |
| *Barriers to action* |  |
| Dysfunctional hypotheses on / about the cause | ‘She has another kind of figure perhaps, or bone structure, just like her father, a little robust, but not overweight.’ |
| Missing / lack of readiness to change the daily structures | ‘...what we like to do [in this region of the country] is one pretzel here, one pretzel there ... because it’s easy. ... And they [the pretzels] are often not counted.’ |
| Being satisfied with current (not sufficient) approach | ‘She did not have too much weight. She was ... only her belly was a little... it shows a little. But it was not a big deal. We did not do much.’ |
| Low level of suffering | ‘And he himself does not think he is an overweight child.’ |
| Postponement to the future | ‚Not yet. She is still in kindergarten. But I think it will be a topic in the future.’ |
| Guilt and shame | ‘That... there is an inhibition threshold. You think to yourself, okay, I am not able to feed my child the right things or I am not able to feed my child the things to not make it overweight.’ |
| Lack of time | ‘If you work full-time, the whole day, you have very little time... It is also a matter of time to observe your child precisely and what it eats.’ |
| Lack of financial resources | ‘There are already many problems with working and costs and all sorts of things.’ |
| Need for autonomy without help by others | ‘I don’t think I would get new insight if I would consult with someone or somewhere.’ |
| Desire to be supported by others | ‘I would expect our pediatrician to help us and if he doesn’t know how, that he refers us to the right place.’ |
| Lack of consequence | ‘I am conscious about nutrition, but I am not that strict, not really strict. And concerning my child, I am roughly strict but not 100%.’ |
| Lack of creativity | ‘Even I am out of ideas sometimes what to prepare for dinner besides bread, making something my child likes that is healthy at the same time. It’s not always easy to find something when your child doesn’t like everything.’ |
| Dysfunctional approach | ‘They always get candy at their grandparents’ house. [Name of child] has a sensitive stomach, if she really eats too much candy it happens that she throws up. […] I asked my father to stop doing this. Or giving her just one piece.’ |
| Child protection | ‘Interviewer: Has he [the pediatrician] tried to explain [daughter] the association between weight and nutrition before? Mother: Not directly, but I would have stopped that anyway.’ |
| Experiences of stigmatization | ‘There are certainly situations in which one says: ‘I am not allowed to join the game’ ... She has told me that repeatedly.’ |
| Social environment is perceived as hindering | ‘I visit the playground with my kids and there is the ice cream shop right around the corner.’ |
| Evaluation of physical and mental health | ‘When I tell her, you can only eat this or that or you can only eat that much…I think that will make it even harder for the child and at her age, it really puts a strain on her.’ |
| Infrastructure is perceived as detaining | ‘We live on the 4^th^ floor, so when she gets a little older and has the confidence, she can go downstairs alone and play in the yard, but right now it’s not really practical.’ |
| Fatalism | ‘And there is nothing you can do.’ |
| *Influenced by...* |  |
| Others | ‘If I listen to my mother-in-law, there is always the same comment: He/she will grow out of it, this is not bad at all. It is obvious for her, if a baby has some ‘baby fat’, it is great and healthy.’ |
| Pediatrician | ‘He [the pediatrician] only said he [the child] is around the [weight] limit. ‘ |
| Childcare / daycare | ‘I think in a kindergarten, in which 20 children are in one group, you cannot expect the kindergarten teacher to know how much your child has eaten.’ |

*Notes.* Quotes were translated from German (original recording) to English.
